# Impact of Bowel Cleansing on Polyp and Adenoma Detection Rate: Post-Hoc Analysis of a Randomized Clinical Trial

**DOI:** 10.3390/cancers17091421

**Published:** 2025-04-24

**Authors:** Marcello Maida, Roberto Vassallo, Alessandro Vitello, Angelo Zullo, Ludovica Venezia, Antonio Facciorusso

**Affiliations:** 1 Department of Medicine and Surgery, University of Enna ‘Kore’, 94100 Enna, Italy; alessandro.vitello@unikore.it; 2 Gastroenterology Unit, Umberto I Hospital, 94100 Enna, Italy; 3 Gastroenterology Unit, Buccheri la Ferla Hospital, 90123 Palermo, Italy; vassallo.roberto@fbfpa.it; 4 Gastroenterology Unit, Nuovo Regina Margherita Hospital, 00153 Roma, Italy; angelomario.zullo@aslroma1.it; 5 Gastroenterology Unit, AOU Maggiore della Carità, 28100 Novara, Italy; ludovica.venezia@maggioreosp.novara.it; 6 Department of Experimental Medicine, Università del Salento, 73100 Lecce, Italy; antonio.facciorusso@virgilio.it; 7 Clinical Effectiveness Research Group, University of Oslo, 0313 Oslo, Norway

**Keywords:** colonoscopy, bowel preparation, cleansing, polyethylene glycol, adenoma detection rate, colorectal cancer

## Abstract

Objectives: To assess the impact of bowel cleansing quality on polyp detection rate (PDR) and adenoma detection rate (ADR) and explore predictors of lesion detection rate in patients undergoing colonoscopy. Methods: This is a post-hoc analysis of a multicenter randomized controlled trial (RCT) comparing 1L polyethylene glycol plus ascorbate (1L PEG+ASC) vs. 4L PEG as bowel preparation for colonoscopy. Results: PDR was significantly higher (35.6% vs. 18.5%, *p* = 0.013), and ADR was higher even if not significantly (25.6% vs. 16.7%, *p* = 0.153) in patients with Boston Bowel Preparation Scale (BBPS) ≥6 over BBPS <6. Comparing patients with BBPS = 9 over BBPS = 7–8, no significant differences were found in PDR (34.5% vs. 38.4%, *p* = 0.483) nor ADR (24.1% vs. 27.2%, *p* = 0.553). At multivariable regression analysis, older age (OR = 1.042, 95%CI = 1.021–1.063; *p* < 0.001), shorter intubation time (OR = 0.891, 95%CI = 0.816–0.972; *p* = 0.010), higher withdrawal time (OR = 1.171, 95%CI = 1.094–1.253; *p* < 0.001) and full consumption of the first dose (OR = 8.368, 95%CI = 1.025–68.331; *p* = 0.047) were independently associated with ADR. Conclusions: This post-hoc analysis of a RCT showed that excellent cleansing (BBPS = 9) over high-quality cleansing (BBPS = 7–8) does not significantly improve PDR or ADR. Neither cleansing success nor preparation types were independently associated with ADR. Compliance with bowel preparation, timing of colonoscopy and withdrawal time are key elements for adequate ADR with potential implications for reducing interval colorectal cancer.

## Simple Summary

In this study we investigated whether the quality of bowel preparation for colonoscopy impacts the detection rate of polyps and adenomas. We analyzed data from our previous clinical trial comparing two different bowel solutions for colonoscopy (1L PEG+ASC vs. 4L PEG) to determine if high-quality or excellent bowel cleansing leads to better polyp detection. Our aim was to identify the optimal level of bowel cleansing and other factors influencing lesion detection rate. These findings may refine colonoscopy best practices, tailoring bowel preparation. In our study neither cleansing success nor preparation types were associated with adenoma detection rate (ADR). On the other side, compliance with bowel preparation, timing of colonoscopy and withdrawal time are key elements for adequate ADR with potential implications for reducing interval colorectal cancer.

## 1. Introduction

Screening has a key role in diagnosis of colorectal cancer (CRC) and early detection of the neoplasm is associated with an improvement in patient survival. Colonoscopy is one of the most widely adopted tests for CRC screening, allowing reduction of disease incidence and mortality [1,2]. Adenoma detection rate (ADR) is a robust indicator independently associated both with the risk of interval colorectal cancer (I-CRC) and death [3,4]. Nevertheless, the detection of colorectal lesions strictly depends on specific quality measures of lower endoscopy [5]. Among these, adequate bowel cleansing is crucial for reliable adenoma detection since it improves visualization of the colorectal mucosa. Nevertheless, the proper threshold of cleansing to ensure clinically relevant outcomes is not clear. According to the American Society for Gastrointestinal Endoscopy (ASGE) and American College of Gastroenterology (ACG) joint task force, the level of bowel cleansing is defined as adequate if it allows the detection of polyps > 5 mm in size [6]. On the other hand, the European Society of Gastrointestinal Endoscopy (ESGE) guidelines define as adequate a colonoscopy with Boston Bowel Preparation Scale (BBPS) ≥ 6 (with a BBPS ≥2 in each segment) or Ottawa Scale ≤ 7 [7]. Beyond adequacy, it is reasonable to assume that a greater quality of cleansing (e.g., overall BBPS score ≥ 7, or segmental BBPS = 3) may further improve the detection of smaller lesions. On this line, recent evidence showed that high-quality over adequate cleansing allows for further improvement in the ADR [8,9] and sessile serrated polyp detection rate (PDR) [10]. Despite the relevance of these results, a subsequent study did not confirm these data [11]. Moreover, predictors of lesion detection rate, other than the adequate quality of bowel cleansing, are not fully known. To address this, we conducted a post-hoc analysis of a randomized clinical trial (RCT) to assess the impact of bowel cleansing quality on PDR and ADR and to explore predictors of lesion detection rate.

## 2. Materials and Methods

### 2.1. Study Design and Participants

This is a post-hoc analysis of prospectively collected data from a phase-IV RCT performed across 10 Italian centers [12]. In that study, male and female in and outpatients aged 18 to 85 years undergoing a screening, surveillance or diagnostic colonoscopy after a preparation with 1L polyethylene glycol plus ascorbate (1L PEG+ASC) or 4L PEG solution were randomized 1:1 to receive 1L PEG+ASC or 4L PEG. Each patient received oral and written information on the preparation to be performed as either afternoon–morning or same-day fashion, completing the consumption within 5 h of the colonoscopy time [13]. The study was observer-blind, as endoscopists were not aware of the preparation assigned to the patient and were not allowed to perform any activities that could interfere with the randomization process.

Conscious sedation and analgesia were used according to the standard clinical practice of each center. Colonoscopy was performed using standard high-definition endoscopes with water jet function. The exam was considered complete if the cecum was reached. All detected lesions were measured using as a reference the open biopsy forceps and classified according to size, morphology, and location.

All colonoscopies were performed by expert endoscopists with >5000 total colonoscopies, at least 500 in the last year, and ADR > 25%.

Full methods regarding colonoscopy procedures are provided in the full paper of the original RCT [12].

### 2.2. Assessments and Measurement

The primary endpoint was to assess the impact of cleansing quality on PDR and ADR. The secondary endpoint was to identify independent predictors of lesion detection in the overall study population and the subgroup for screening and surveillance.

The quality of bowel preparation was evaluated through the BBPS by treatment-blinded site endoscopists after specific training. The BBPS scores of all segments were rated after the required washing and suctioning during withdrawal phase of colonoscopy and ranged in four grades (0 = inadequate, 1 = fair, 2 = good, 3 = excellent) and added as the total BBPS score, from 0 to 9 [14].

Cleansing success (CS) was defined as a BBPS ≥ 6 with a segmental BBPS ≥ 2, high-quality cleansing (HQC) as a total BBPS = 7–8, and excellent cleansing (EC) as a total BBPS = 9 or a segmental BBPS = 3.

PDR and ADR were defined as the percentage of patients with at least one polyp or adenoma on colonoscopy out of the total patients included in the study, respectively.

Adenoma per colonoscopy (APC) was defined as the number of adenomas detected out of the total number of colonoscopies.

### 2.3. Statistical Analysis

Continuous variables were reported as mean ± standard deviation, and categoric variables were summarized as frequency and percentage. Comparisons of variables were made by *t*-test, Chi-square test and Fisher’s exact test as appropriate. A *p*-value of less than 0.05 was considered to indicate statistical significance. Multivariable logistic regression models were designed to identify the variables independently associated with PDR and ADR. All variables included in the models were selected through stepwise model selection and were guided by clinical relevance. Corrections for multiple testing were not applied when fitting multiple logistic regression models, as this approach can be overly conservative. Statistical analysis was performed using SPSS v. 28.0 for Macintosh (SPSS Inc., Chicago, IL, USA).

## 3. Results

Of the 478 eligible patients, 433 were analyzed for efficacy (220 randomized to 1L PEG+ASC and 213 to the 4L PEG arm). Mean age was 59.3 ± 14.9, 49.6% of patients were males and 59.4% were outpatients. Overall, 65.5% of patients performed colonoscopy for diagnostic and 34.5% for screening or surveillance purposes. Concerning characteristics of the colonoscopy procedures, 68.6% performed an afternoon–morning and 31.4% a same-day preparation regimen, cecal intubation rate was 96.2% and average withdrawal time was 9.9 ± 4.1 min.

### 3.1. Type and Morphology of Lesions Detected

Overall, 221 lesions were found. In detail, 156 were adenomas (of these, 80 were advanced adenomas), and 65 were not adenomatous polyps (Table 1). No significant difference in the detection of overall lesions and adenomas was found across the two treatment arms. Among adenomas, the rate of sessile, pedunculated and flat lesions was 56.5%, 16.0% and 27.5% in the 4L PEG arm, and 58.8%, 17.6% and 23.6% in the 1L PEG+ASC arm, respectively. The location of adenomas was proximal and rectosigmoid in 71.8% and 28.2% in the 4L PEG arm, 77.6% and 22.4% in the 1L PEG+ASC arm, respectively (*p* = 0.403). A significant difference was observed in the detection of non-adenomatous polyps across the two study arms, since a higher rate of flat lesion was found in the 1L PEG+ASC compared to 4L PEG (41.2% vs. 16.7%, OR 3.50, 95%CI = 1.07–11.27, *p* = 0.032).

### 3.2. Lesion Detection Rate by Quality of Bowel Cleansing

**Figure 1:**
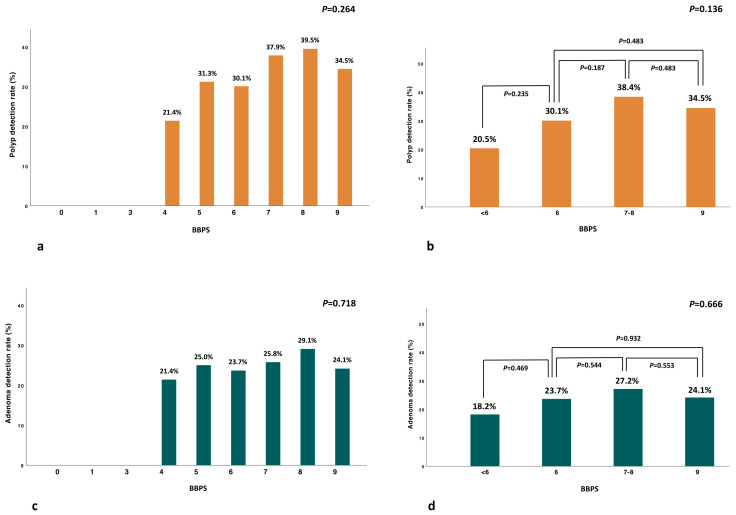
Percentages of PDR (orange) (**a**,**b**) and ADR (green) (**c**,**d**) by BBPS categories.

The analysis of PDR and ADR by BBPS value showed a nonsignificant trend in PDR and ADR for BBPS > 6 (Figure 1a,c). PDR was significantly higher in patients with CS compared to those without CS (35.6% vs. 18.5%, *p* = 0.013) (Table 2), and similar in patients with EC (BBPS = 9) over HQC (BBPS = 7–8) (34.5% vs. 38.4%, *p* = 0.483) (Figure 1b). 

ADR was higher, even if not statistically significant, in patients with CS compared to those without CS (25.6% vs. 16.7%, *p* = 0.153) (Table 2) and comparable in patients with EC over HQC (24.1% vs. 27.2%, *p* = 0.553) (Figure 1d). Similar results were confirmed in the subgroup analysis comparing EC (BBPS = 9) versus the absence of EC (BBPS = 6–8), showing similar PDR (34.5% vs. 36.3%, *p* = 0.716) and ADR (24.1% vs. 26.5%, *p* = 0.609), respectively. Of note, no lesions were detected in patients with BBPS < 4 (Figure 1a,c). 

Finally, APC was significantly higher in patients with CS compared to those without CS (1.47 ± 0.78 vs. 1.00 ± 0.0, *p* < 0.001), but no difference was found between EC over HQC (1.36 ± 0.76 vs. 1.52 ± 0.83, *p* = 0.374).

### 3.3. Lesion Detection Rate by Colonic Segments

**Figure 2:**
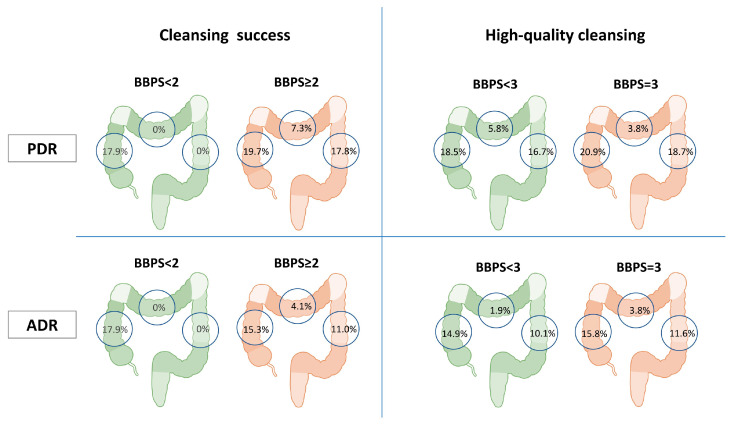
Segmental PDR and ADR by BBPS score.

A subgroup analysis was performed to assess the impact of the segmental cleansing on the lesion detection rate of the same segment (Table 2, Figure 2). The right colon PDR (19.7% vs. 17.9%, *p* = 0.813) and ADR (15.3% vs. 17.9%, *p* = 0.723) were similar in patients with segmental BBPS ≥ 2 over BBPS < 2. Along the same line, PDR (20.9% vs. 18.5%, *p* = 0.541) and ADR (15.8% vs. 14.9%, *p* = 0.796) were similar in patients with segmental BBPS = 3 over BBPS = 2.

Moreover, the transverse colon PDR (7.3% vs. 0%, *p* = 0.779) and ADR (4.1% vs. 0%, *p* = 0.835) were similar in patients with segmental BBPS ≥ 2 vs. BBPS < 2. Similarly, PDR (3.8% vs. 5.8%, *p* = 0.686) and ADR (3.8% vs. 1.9%, *p* = 0.540) were comparable in patients with segmental BBPS = 3 over BBPS = 2.

Finally, the left colon PDR (17.8% vs. 0%, *p* = 0.641) and ADR (11.0% vs. 0%, *p* = 0.725) were similar in patients segmental BBPS ≥ 2 vs. BBPS < 2. In this case also, PDR (18.7% vs. 16.7%, *p* = 0.602) and ADR (11.6% vs. 10.1%, *p* = 0.634) did not differ in patients with segmental BBPS = 3 over BBPS = 2.

### 3.4. Lesion Detection Rate by Type of Bowel Preparation

The 1L PEG+ASC showed a higher CS rate (91.8% vs. 83.6%; OR = 2.207; 95%CI: 1.2–4.0; *p* = 0.009) and EC (39.1% vs. 27.7, *p* = 0.001) as compared to the 4L PEG preparation, respectively. Despite the higher efficacy of 1L-PEG+ASC, no differences in PDR (34.1% vs. 32.9%, *p* = 0.787), ADR (25.5% vs. 23.5%, *p* = 0.632), and APC (1.5 ± 0.8 vs. 1.4 ± 0.7, *p* = 0.617) were found in patients undergoing 1L PEG+ASC and 4L PEG, respectively.

### 3.5. Predictors of Lesion Detection Rate

At multivariable logistic regression analysis, older age (OR = 1.040, 95%CI = 1.023–1.057; *p* < 0.001), CS (OR = 2.250, 95%CI = 1.009–5.020; *p* = 0.048) and higher withdrawal time (OR = 1.155, 95%CI = 1.079–1.236; *p* < 0.001) were independently associated with PDR (Table 3). On the other hand, older age (OR = 1.042, 95%CI = 1.021–1.063; *p* < 0.001), shorter intubation time (OR = 0.891, 95%CI = 0.816–0.972; *p* = 0.010), higher withdrawal time (OR = 1.171, 95%CI = 1.094–1.253; *p* < 0.001) and full consumption of the first dose (OR = 8.368, 95%CI = 1.025–68.331; *p* = 0.047) were independently associated with ADR (Table 4). In the subgroup of patients undergoing colonoscopy for screening or surveillance (N = 220) longer preparation time (OR = 1.559, 95%CI = 1.161–2.093; *p* = 0.003), and colonoscopy within 5 h after preparation (OR = 3.119, 95%CI = 1.013–9.608; *p* = 0.047) were independently associated with ADR (Supplementary Table S1).

## 4. Discussion

Optimal colon cleansing is necessary for the proper detection of lesions [7]. Nevertheless, the role of excellent versus sufficient cleaning in increasing the lesion detection rate remains controversial. A post-hoc analysis of three RCTs showed that excellent over adequate cleansing may be associated with a further increase in lesion detection rate [8]. In contrast, a recent retrospective study showed no difference between excellent and good bowel preparation quality in improving PDR and ADR [11]. 

The results of this post-hoc analysis of a large multicenter RCT showed that PDR and APC were significantly higher, and ADR was higher, even if not significantly, in patients with CS compared to those without. Nevertheless, no significant differences in PDR, ADR and APC were found when comparing EC over HQC. To avoid confounding due to the cumulative cleansing and detection rate data on the entire colon, we also analyzed the lesion detection rate by colonic segment in relation to the cleansing of the same segment without finding significant differences. 

These results contrast with previous data showing an advantage in lesion detection rate for excellent over adequate cleansing [8,9]. The possible explanation of this phenomenon is that the quality of bowel cleansing contributes to the detection of lesions up to a certain threshold, beyond which the skill of the operator plays a crucial role. In support of this hypothesis, the baseline ADR (>25%) of endoscopists participating in the study may justify the absence of a further gain in lesion detection in the presence of improved colon cleansing. 

As discussed above, we evaluated the bowel cleansing quality as a proxy for lesion detection rate, regardless of the preparation used. Nevertheless, in the absence of a central reading, the measurement of colon cleansing may be affected by interobserver variability. Therefore, we directly analyzed the impact of bowel preparation type on lesion detection rate, bypassing the bowel cleansing measurement and its potential biases. The Over-2019 RCT showed that 1L PEG+ASC overcame 4L PEG in achieving CS and EC [12]. Nevertheless, the results of this post-hoc analysis failed to demonstrate differences in PDR or ADR between the two preparations, independent of the grade of cleansing. This data is in line with those of a similar RCT comparing 1L PEG+ASC versus 4L PEG [15], and of a meta-analysis of 9 RCTs assessing the efficacy of 1L PEG+ASC versus all other bowel preparations [16]. Both studies showed no significant difference in ADR despite a higher efficacy of 1L PEG+ASC over the comparators. 

Moreover, we have also performed multivariable logistic regression models to explore the possible factors independently associated with lesion detection rate, showing that neither CS nor EC was independently associated with ADR. Other technical factors were also shown to have a crucial role in lesion detection. Among these, a shorter intubation time, possibly reflecting higher operator skill, and a higher withdrawal time were independently associated with ADR. This confirms the results of the previous study [17] and corroborates that proper exposure and view of the colonic mucosa are essential for lesion detection. Of note, full adherence to the assumption of the preparation was independently associated with ADR. This shows that compliance strongly impacts ADR, regardless of the type of solution consumed. 

This study has several strengths. The analysis was performed on data from an RCT, including a large number of centres, with a standardized cleansing quality assessment. This guarantees a high reproducibility of the results. Furthermore, we evaluated the lesion detection rate (PDR, ADR) as well as the APC, ensuring both a per-patient and a per-polyp perspective. Finally, compared to previous studies, we evaluated the association between bowel cleansing and lesion detection rate both overall and per segment, excluding a possible confounding related to the score aggregation, finding no differences for each segment. 

Nonetheless, we must also acknowledge some limitations. First of all, the RCT from which this analysis was carried out was designed to assess the efficacy of bowel cleansing and, therefore, it is underpowered to evaluate ADR consistently. Second, the absence of a central reading for the assessment of bowel cleansing and histological examination did not exclude inter-observer variability. Third, the study included only patients receiving 1L PEG+ASC or 4L PEG preparation. Therefore, this analysis excludes the possible effect of 2L PEG preparations.

## 5. Conclusions

In conclusion, this post-hoc analysis of a large RCT showed that EC (BBPS = 9) over HQC (BBPS = 7–8) does not significantly improve PDR or ADR, not confirming the findings of a previous report [8]. In addition, neither cleansing success nor preparation types were independently associated with ADR. Compliance with bowel preparation, timing of colonoscopy and accurate withdrawal are key elements.

## Figures and Tables

**Table 1:** Morphology of detected adenomas and location of detected polyps.

	4L PEG	1L PEG+ASC	Odds Ratio (CI-95%)	*p* Value
**Morphology of polyps**				
**Adenoma**				0.843
- Pedunculated	11 (16.0%)	15 (17.6%)	1	
- Sessile	39 (56.5%)	50 (58.8%)	0.94 (0.38–2.27)	
- Flat	19 (27.5%)	20 (23.6%)	0.77 (0.28–2.09)	
Missing values	2	0		
**Non-adenomatous polyps**				**0.032**
- Sessile	25 (83.3%)	20 (58.8%)	1	
- Flat	5 (16.7%)	14 (41.2%)	3.50 (1.07–11.27)	
Missing values	0	1		
**Location of polyps**				
**Adenoma**				0.403
- Proximal ^†^	51 (71.8%)	66 (77.6%)	1	
- Rectosigmoid	20 (28.2%)	19 (22.4%)	0.73 (0.35–1.51)	
**Non-adenomatous polyps**				0.717
- Proximal ^†^	19 (63.3%)	23 (67.6%)	1	
- Rectosigmoid	11 (36.7%)	11 (32.4%)	0.82 (0.29–2.32)	
Missing values	0	1		
**Overall**				
- Total number of polyps	101	120		
- Pedunculated	11 (11.1%)	15 (12.6%)	1	0.677
- Sessile	64 (64.6%)	70 (58.8%)	0.80 (0.34–1.87)	
- Flat	24 (24.2%)	34 (28.6%)	1.03 (0.40–2.65)	
- Proximal ^†^	70 (69.3%)	89 (74.8%)	1	0.365
- Rectosigmoid	31 (30.7%)	30 (25.2%)	0.76 (0.42–1.37)	

Analysis limited to non-neoplastic lesions, with histology available. Abbreviations: CI, Confidence interval; PEG, polyethylene glycol; PEG+ASC, polyethylene glycol plus ascorbate. The odds ratio compares the odds of the polyp characteristics among 1L PEG+ASC patients with the odds of the polyp characteristics among 4L PEG patients. ^†^ Proximal is defined as descending colon, transverse colon, ascending colon, or cecum.

**Table 2:** Overall lesion detection rate by quality of colon cleansing.

	Polyp Detection Rate (PDR)						Adenoma Detection Rate (ADR)					
	Cleansing Success			High-Quality Cleansing			Cleansing Success			High-Quality Cleansing		
	BBPS < 6	BBPS ≥ 6 *	*p*	BBPS = 7–8	BBPS = 9	*p*	BBPS < 6	BBPS ≥ 6 *	*p*	BBPS = 7–8	BBPS = 9	*p*
**Overall colon**	18.5%	35.6%	**0.013**	38.4%	34.5%	0.483	16.7%	25.6%	0.153	27.2%	24.1%	0.553
	**Cleansing success**			**High-quality cleansing**			**Cleansing success**			**High-quality cleansing**		
**Segmental colon**	**BBPS < 2**	**BBPS ≥ 2**	***p***	**BBPS < 3**	**BBPS = 3**	***p***	**BBPS < 2**	**BBPS ≥ 2**	***p***	**BBPS < 3**	**BBPS = 3**	***p***
**-Right colon**	17.9%	19.7%	0.813	18.5%	20.9%	0.541	17.9%	15.3%	0.723	14.9%	15.8%	0.796
**-Transverse colon**	0%	7.3%	0.779	5.8%	3.8%	0.686	0%	4.1%	0.835	1.9%	3.8%	0.540
**-Left colon**	0%	17.8%	0.641	16.7%	18.7%	0.602	0%	11.0%	0.725	10.1%	11.6%	0.634

* BBPS ≥ 6 with segmental BBPS ≥ 2.

**Table 3:** Univariable and Multivariable logistic regression analysis of variables associated with PDR.

	Polyp Detection Rate (PDR)				
	Univariable Analysis			Multivariable Analysis	
Variable	No Polyp Detected (N = 288)	Polyp Detected (N = 145)	*p* Value	Odds Ratios (CI-95%)	*p* Value
**Age**	56.8 ± 15.9	64.1 ± 11.2	<0.001	1.040 (1.023–1.057)	**<0.001**
**Preparation regimen**					
-Afternoon morning	186 (64.6%)	111 (76.6%)	0.011	1.602 (0.984–2.606)	0.058
-Same-day	102 (35.4%)	34 (23.4%)			
**Cleansing success**	244 (84.7%)	136 (93.8%)	0.077	2.250 (1.009–5.020)	**0.048**
**Cecal intubation**	275 (95.5%)	144 (99.3%)	0.034	4.584 (0.509–41.330)	0.175
**Withdrawal time**	9.0 ± 3.1	11.1 ± 4.3	<0.001	1.155 (1.079–1.236)	**<0.001**

**Table 4:** Univariable and Multivariable logistic regression analysis of variables associated with ADR.

	Adenoma Detection Rate (ADR)				
	Univariable Analysis			Multivariable Analysis	
Variable	No Adenoma Detected (N = 327)	Adenoma Detected (N = 106)	*p* Value	Odds Ratios (CI-95%)	*p* Value
**Age**	57.4 ± 15.3	64.8 ± 11.8	<0.001	1.042 (1.021–1.063)	**<0.001**
**Diabetes**	37 (11.3%)	20 (18.9%)	0.046	1.035 (0.522–2.051)	0.923
**Preparation regimen**					
- Afternoon-morning	215 (65.7%)	82 (77.4%)	0.025	1.668 (0.957–2.907)	0.071
- Same-day	112 (34.3%)	24 (22.6%)			
**Preparation duration**	2.8 ± 2.0	3.4 ± 3.0	0.018	1.094 (0.999–1.198)	0.051
**Intubation time**	8.7 ± 4.1	7.8 ± 2.8	0.043	0.891 (0.816–0.972)	**0.010**
**Withdrawal time**	9.2 ± 3.2	11.3 ± 4.6	<0.001	1.171 (1.094–1.253)	**<0.001**
**Compliance with bowel preparation (I dose)**					
- 100% intake of overall volume	308 (94.2%)	105 (99.1%)	0.038	8.368 (1.025–68.331)	**0.047**
- <100% intake of overall volume	19 (5.8%)	1 (0.9%)			

## Data Availability

The original contributions presented in this study are included in the article/Supplementary Materials. Further inquiries can be directed to the corresponding author.

## Supplementary Materials

The following supporting information can be downloaded at: https://www.mdpi.com/2072-6694/17/9/1421/s1, Table S1: Univariable and Multivariable logistic regression analysis of variable associated with ADR in patients undergoing screening or surveillance.

## Abbreviations

The following abbreviations are used in this manuscript:

CRC	Colorectal Cancer
ADR	Adenoma Detection Rate
I-CRC	Interval Colorectal Cancer
ASGE	American Society for Gastrointestinal Endoscopy
ACG	American College of Gastroenterology
ESGE	European Society of Gastrointestinal Endoscopy
BBPS	Boston Bowel Preparation Scale
RCT	Randomized Clinical Trial
PEG	Polyethylene Glycol
ASC	Ascorbate
PEG+ASC	Polyethylene Glycol plus Ascorbate
PDR	Polyp Detection Rate
CS	Cleansing Success
HQC	High-Quality Cleansing
EC	Excellent Cleansing
APC	Adenoma Per Colonoscopy
OR	Odds Ratio
CI	Confidence Interval
