# Supplementary material for: Impact of Bowel Cleansing on Polyp and Adenoma Detection Rate: Post-Hoc Analysis of a Randomized Clinical Trial
Source: Cancers (Basel). 2025 Apr 24;17(9):1421. doi: 10.3390/cancers17091421 (PMC12071129; doi:10.3390/cancers17091421)
Supplement: Supplementary file 1 [file cancers-17-01421-s001.zip › cancers-3575321-supplementary.pdf]

Supplementary Table S1. Univariable and Multivariable logistic regression analysis of variable associated with ADR in patients undergoing screening or surveillance.

	Univariable analysis			Multivariable analysis	
**Variable**	**No adenoma detected (N=112)**	**Adenoma detected (N=39)**	**P value**	**Odds Ratios (CI-95%)**	**P value**
**Age**	60.7 ± 12.1	65.2 ± 9.0	0.037	1.015 (0.974-1.058)	0.475
**Preparation duration**	2.4 ± 1.2	3.7 ± 3.2	0.025	1.559 (1.161-2.093)	**0.003**
**Colonoscopy within 5h after preparation**	59 (62.8%)	26 (83.9%)	0.029	3.119 (1.013-9.608)	**0.047**
**Withdrawal time**	9.5 ± 4.2	11.7 ± 5.7	0.035	1.054 (0.970-1.146)	0.217
